# External Validation of the Phoenix Sepsis Score in Children With Suspected Community-Acquired Sepsis

**DOI:** 10.1001/jamanetworkopen.2025.1412

**Published:** 2025-03-21

**Authors:** Elliot Long, Meredith L. Borland, Shane George, Shefali Jani, Eunicia Tan, Natalie Phillips, Amit Kochar, Simon Craig, Anna Lithgow, Arjun Rao, Stuart Dalziel, Ed Oakley, Stephen Hearps, Ben Gelbart, Sarah McNab, Fran Balamuth, Scott L. Weiss, Nathan Kuppermann, Charlotte Brad, Amanda Williams, Franz E. Babl

**Affiliations:** 1 Department of Emergency Medicine, The Royal Children’s Hospital, Parkville, Victoria, Australia; 2 Clinical Sciences, Murdoch Children’s Research Institute, Parkville, Victoria, Australia; 3 Department of Paediatrics, Faculty of Medicine, Dentistry, and Health Sciences, University of Melbourne, Parkville, Victoria, Australia; 4 Department of Critical Care, Faculty of Medicine, Dentistry, and Health Sciences, University of Melbourne, Parkville, Victoria, Australia; 5 Department of Emergency Medicine, Perth Children’s Hospital, Nedlands, Western Australia, Australia; 6 Divisions of Emergency Medicine and Paediatrics, School of Medicine, University of Western Australia, Perth, Western Australia, Australia; 7 Department of Emergency Medicine and Children’s Critical Care, Gold Coast University Hospital, Southport, Queensland, Australia; 8 School of Medicine and Dentistry, Griffith University, Southport, Queensland, Australia; 9 Child Health Research Centre, The University of Queensland, Brisbane, Queensland, Australia; 10 Department of Emergency Medicine, The Children’s Hospital at Westmead, Westmead, New South Wales, Australia; 11 Faculty of Medicine and Health, University of Sydney, Camperdown, New South Wales, Australia; 12 Middlemore Hospital, Aukland, New Zealand; 13 Department of Surgery and Paediatrics: Child and Youth Health, The University of Auckland, Auckland, New Zealand; 14 Emergency Department, Queensland Children’s Hospital, South Brisbane, Queensland, Australia; 15 Child Health Research Centre, University of Queensland, Queensland, Australia; 16 Department of Emergency Medicine, Women and Children’s Hospital, Adelaide, South Australia, Australia; 17 Department of Acute Care Medicine, University of Adelaide, Adelaide, South Australia, Australia; 18 Paediatric Emergency Department, Monash Medical Centre, Monash Health, Victoria, Australia; 19 Department of Paediatrics, School of Clinical Sciences, Monash University, Clayton, Victoria, Australia; 20 Department of Paediatrics, Royal Darwin Hospital, Tiwi, Northern Territory, Australia; 21 Department of Emergency Medicine, Sydney Children’s Hospital, Randwick, New South Wales, Australia; 22 School of Women’s and Children’s Health, University of New South Wales, Kensington, New South Wales, Australia; 23 Children’s Emergency Department, Starship Children’s Hospital, Auckland, New Zealand; 24 Paediatric Intensive Care Unit, The Royal Children’s Hospital, Parkville, Victoria, Australia; 25 Department of General Medicine, The Royal Children’s Hospital, Parkville, Victoria, Australia; 26 Department of Pediatrics, University of Pennsylvania Perelman School of Medicine, Philadelphia; 27 Division of Emergency Medicine, Children’s Hospital of Philadelphia, Philadelphia, Pennsylvania; 28 Nemours Children’s Health and Sydney Kimmel Medical College at Thomas Jefferson University, Philadelphia, Pennsylvania; 29 Departments of Pediatrics and Emergency Medicine, the George Washington School of Medicine and Health Sciences, and Children’s National Hospital, Washington, DC

## Abstract

**Importance:**

The novel Phoenix Sepsis Score and sepsis criteria were derived and validated using a multicountry dataset and proposed as a new definition for sepsis in children.

**Objective:**

To externally validate the Phoenix Sepsis Score and sepsis criteria in a cohort of children hospitalized with suspected community-acquired sepsis.

**Design, Setting, and Participants:**

This diagnostic study used data from the multicenter, multicountry Sepsis Epidemiology in Australian and New Zealand Emergency Departments (SENTINEL) study, collected from 2021 to 2023 and including 90-day follow-up. Children admitted to the hospital through 11 emergency departments in Australia and New Zealand and treated with parenteral antibiotics with either (1) a provisional diagnosis of sepsis or (2) treatment for sepsis (intravenous fluid bolus to treat poor perfusion) were included.

**Exposure:**

Development of organ dysfunction over the first 24 hours of hospitalization.

**Main Outcomes and Measures:**

The main outcomes were (1) in-hospital mortality and (2) death or requirement for extracorporeal life support (ECLS) within 72 hours of hospitalization.

**Results:**

A total of 6232 children were included in the analysis, with a median (IQR) age of 2.1 (0.3-7.1) years, 3386 (54.1%) male, in-hospital mortality of 60 (1.0%), and death or ECLS within 72 hours in 36 (0.6%). In this population, the worst Phoenix Sepsis Score calculated over the first 24 hours of hospitalization had an area under the precision recall curve of 0.17 (95% CI, 0.07-0.28) for predicting in-hospital mortality and 0.23 (95% CI, 0.11-0.36) for predicting death or ECLS within 72 hours. Overall, 306 children (4.9%) met the Phoenix sepsis criteria, of whom 33 (10.8%) died in the hospital (nearly half of the total number who died) and 28 (9.2%) died or required ECLS within 72 hours. The Phoenix sepsis criteria had a sensitivity of 55.0% (95% CI, 41.6%-67.9%) and positive predictive value (PPV) of 10.8% (95% CI, 7.6%-14.9%) for in-hospital mortality and sensitivity of 77.8% (95% CI, 60.8%-89.9%) and PPV of 9.2% (95% CI, 6.2%-13.0%) for death or ECLS within 72 hours. Coagulation data for the calculation of the Phoenix Sepsis Score were missing in more than 85% of children.

**Conclusions and Relevance:**

In this multicenter diagnostic study of children hospitalized with suspected sepsis, the Phoenix Sepsis Score and sepsis criteria had similar performance to the original derivation and validation cohorts. The small proportion of children meeting Phoenix sepsis criteria, missingness of data, timing of application, and lack of sensitivity for in-hospital mortality limit the clinical utility of the criteria.

## Key Points

**Question:**

How do the Phoenix Sepsis Score and sepsis criteria perform in an external dataset?

**Findings:**

This diagnostic study that included 6232 children with suspected community-acquired sepsis found that the test characteristics of the Phoenix Sepsis Score and sepsis criteria had similar performance to the original derivation and validation study. However, only a small proportion of patients met the Phoenix sepsis criteria, there was a high proportion of missing data, the Phoenix score was calculated after 24 hours of hospitalization, and almost half of in-hospital deaths did not meet the Phoenix sepsis criteria.

**Meaning:**

These findings suggest that while the Phoenix sepsis criteria will help standardize how sepsis is defined for epidemiological and benchmarking purposes, they may have limited clinical applicability.

## Teaser

This diagnostic study evaluates the performance of the Phoenix Sepsis Score among children admitted to the emergency department in Australia and New Zealand with suspected sepsis.

## Introduction

Pediatric sepsis is a serious global public health problem, affecting more than 25 million children per year and responsible for the death of more than 3 million children per year worldwide.^1^ Efforts to determine sepsis epidemiology, benchmark care, and enroll patients in research studies, however, have been hampered by the lack of a clear sepsis definition and validated criteria for operationalization.

The Systemic Inflammatory Response Syndrome criteria, established through expert elicitation, are neither sensitive nor specific for mortality due to sepsis in children.^2,3^ Organ dysfunction due to suspected or proven infection has been proposed as a better predictor of sepsis mortality.^4^ Through harmonization of electronic health record data from 10 health systems in 5 countries, specific organs and thresholds for dysfunction were derived and validated in retrospectively identified children hospitalized with suspected infections.^5^ A 4-organ model, the Phoenix Sepsis Score, combined derangements in the cardiovascular, neurological, respiratory, and coagulation systems and had a reported area under the precision recall curve (AUPRC) of 0.23 to 0.38 (95% CI, 0.20-0.39) for predicting in-hospital mortality and a reported AUPRC of 0.14 to 0.48 (95% CI, 0.13-0.48) for predicting death or extracorporeal life support (ECLS) within 72 hours. Using a Phoenix Sepsis Score of 2 points or greater as diagnostic criteria for sepsis had an overall sensitivity of 56.5% (95% CI, 54.3%-58.7%) and positive predictive value of 9.9% (95% CI, 9.4%-10.5%) for in-hospital mortality and an overall sensitivity of 68.1% (95% CI, 65.3%-70.8%) and positive predictive value of 6.6% (95% CI, 6.2%-7.0%) for death or ECLS within 72 hours.^5^

We sought to externally validate the Phoenix Sepsis Score in a cohort of children hospitalized with suspected community-acquired sepsis. We applied the Phoenix organ dysfunction model to data collected as part of the Sepsis Epidemiology in Australian and New Zealand Children (SENTINEL) study.^6^ The SENTINEL study was a multicenter cohort study collecting data from children hospitalized with suspected community-acquired sepsis. We report the test characteristics of the Phoenix Sepsis Score and sepsis criteria.

## Methods

### Study Design, Setting, and Population

In this multicenter, multicountry study, data were collected from 11 emergency departments (EDs): 8 tertiary pediatric EDs and 3 large mixed pediatric and adult EDs. All participating sites were in Australia and New Zealand, had annual ED censuses of more than 20 000 children, and were members of the Paediatric Research in Emergency Departments International Collaborative (PREDICT) network.^7^ The annual volume of the 11 participating EDs in total was more than 450 000 childhood (aged 0 to <18 years) presentations. The study was conducted between April 2021 and December 2023, with staggered start dates at some sites due to COVID-19–related delays in obtaining ethics and governance approval (eTable 1 in Supplement 1).

Approval for the conduct of this study was provided by the Royal Children’s Hospital, Melbourne, Australia Human Research Ethics Committee. The Murdoch Children’s Research Institute (MCRI) served as the primary sponsor for this trial. The study operated under a waiver of consent for data extraction from the medical record and informed verbal consent for follow-up contact at the study lead site (with written consent required in some jurisdictions) in compliance with the Australian National Statement on Ethical Conduct in Human Research.^8^ This study was registered with the Australian and New Zealand Clinical Trials Registry on July 15, 2021, prior to commencement of recruitment (ACTRN12621000920897).

All participating sites had governance approvals from their local human research ethics committees for the conduct of the study. The study was reported according to the Standards for Reporting of Diagnostic Accuracy (STARD) guidelines.^9^

### Inclusion and Exclusion Criteria

Children up to 18 years of age who presented to participating EDs with suspected sepsis were included. Suspected sepsis was defined as admission to the hospital for parenteral antibiotics and either (1) a provisional (admission) diagnosis of sepsis, septicemia, or septic shock; or (2) treatment for sepsis, operationalized as treatment with 1 or more fluid boluses (defined as a fixed volume of fluid administered over <30 minutes to treat impaired perfusion) (eTable 2 in Supplement 1). We included treatment for sepsis as an inclusion criteria, as prior studies had identified a substantial proportion of patients with infection requiring intensive care unit (ICU)–level care did not have an admission diagnosis of sepsis.^10^ This was operationalized using the administration of a fluid bolus because this was the initial treatment for sepsis recommended in all guidelines and pathways at participating sites.^11^

Patients not admitted through the ED (such as direct interhospital ICU transfers) and patients who were admitted to another hospital ward prior to ED transfer were excluded due to difficulty obtaining initial organ function data. Patients presenting with trauma were excluded.

### Patient Recruitment, Study Procedures, and Data Collection

Patients were screened by their treating clinicians for eligibility. Enrollment of eligible patients included verbal or written consent (jurisdiction dependent) for permission to contact families for follow-up 90 days from enrollment. Missed eligible patients were identified by the research team at each participating site through real-time reviews of all ED attendances and ICU admission records. Consent was obtained in-person for hospitalized patients. In the rare instance in which the patient was discharged home prior to consent, telephone consent was obtained as soon as possible. Deidentified routinely collected data from the medical records of included patients were extracted and entered into a secure web-based Research Electronic Data Capture (REDCap) database housed at MCRI according to the study protocol.^6^ Ethnicity was recorded as self-reported by the parent or guardian and recorded in the patient medical record. Ethnicity was assessed to evaluate whether Indigenous peoples were overrepresented with respect to sepsis incidence or severity.

### Statistical Analysis

Primary and secondary outcomes were harmonized with the original Phoenix validation study and are listed in the Box. The area under the precision recall curve (AUPRC) was the primary measure used for Phoenix Sepsis Score validation. Secondary measures included the area under the receiver operating characteristics curve (AUROC) of the Phoenix Sepsis Score and the test characteristics of the Phoenix sepsis criteria (Phoenix Sepsis Score ≥2). Outcomes evaluated were in-hospital mortality and either death or requirement for ECLS within 72 hours of hospitalization. Data from the first 24 hours of patient hospitalization were used to determine the Phoenix Sepsis Score, with the most abnormal overall score during this time used for analysis. Pediatric complex chronic conditions were defined by relevant *International Statistical Classification of Diseases and Related Health Problems, Tenth Revision*, diagnoses using the major comorbidities of technological dependence, malignant neoplasm, and transplant, in keeping with the original validation study.^12^ For asynchronously collected data points used in the calculation of the Phoenix Sepsis Score, we used a last-observation-carried-forward approach within physiologically appropriate time windows, in keeping with the original derivation and validation study.^5^ Completely missing values were treated as nonadditive to organ dysfunction scoring in keeping with the original derivation and validation study.^5^ Data from the original Phoenix study were reported for high- and low-resource sites separately. We pooled these data for comparison with the SENTINEL cohort. Analyses were conducted in Stata version 18 (StataCorp).

Box. **Primary and Secondary Outcome Measures**

**Primary Outcome**

Area under the precision recall curve of the Phoenix Sepsis Score for predicting in-hospital mortality

**Secondary Outcomes**

Area under the receiver operating characteristic curve of the Phoenix Sepsis Score for predicting in-hospital mortality
Sensitivity and positive predictive value of Phoenix sepsis criteria for predicting in-hospital mortality
Sensitivity and positive predictive value of Phoenix sepsis criteria for predicting death or extracorporeal life support within 72 hours

## Results

**Figure 1.**
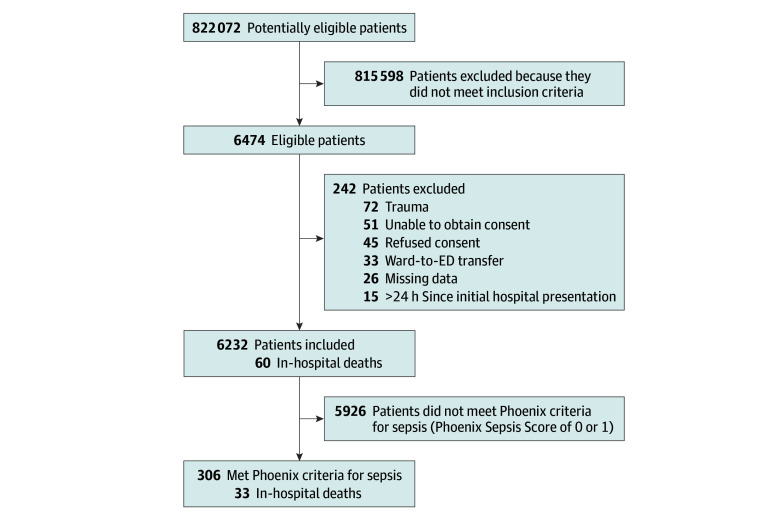
Flow Diagram of Included Participants ED indicates emergency department.

Of 822 072 potentially eligible patients, 6474 were eligible and 6232 were included in the analysis. The median (IQR) age was 2.1 (0.3-7.1) years, and 3386 (54.1%) were male. Overall, 306 children (4.9%) had Phoenix Sepsis Scores of 2 or greater and met the Phoenix sepsis criteria (Figure 1).

Demographic characteristics of the SENTINEL and original Phoenix cohorts are shown in Table 1. Overall, in-hospital mortality in the study population was 60 of 6232 (1.0%) and overall death or ECLS within 72 hours was 36 of 6258 (0.6%). A total of 33 of 60 children who died in hospital (55.0%) and 28 of 36 children who died or required ECLS within 72 hours (77.8%) met the Phoenix sepsis criteria. Patient characteristics by inclusion criteria (provisional diagnosis or sepsis vs treatment for sepsis) are presented in eTable 3 in Supplement 1. Missingness of data used to calculate the Phoenix Sepsis Score are presented in eTable 4 in Supplement 1. For example, more than 85% of children were missing data on coagulation.

**Table 1.** Characteristics of Participants From the SENTINEL and Original Phoenix Cohorts

Characteristic	Participants, No. (%)	
	SENTINEL cohort (n = 6232)	Original Phoenix cohort (n = 172 984)
Age at presentation, median (IQR), y	2.1 (0.3-7.1)	3.7 (0.9-9.4)
Sex		
Female	2830 (45.2)	83 909 (48.5)
Male	3386 (54.1)	89 069 (51.5)
Other	2 (<0.1)	0
Ethnicity		
Indigenous^a^	599 (9.6)	266 (<0.1)
Comorbidities per PCCC		
1	1168 (18.7)	12 566 (8.7)
≥2	1345 (21.5)	35 062 (20.3)
ICU admission	1080 (17.3)	30 968 (17.9)
Outcomes		
In-hospital mortality	60 (1.0)	2065 (1.2)
Death or extracorporeal life support within 72 h	36 (0.6)	1139 (0.6)

Abbreviations: ICU, intensive care unit; PCCC, pediatric complex chronic condition.

^a^ Indigenous ethnicity includes Aboriginal and Torres Strait Islander, American Indian, Alaska Native, Māori, Native Hawaiian, and Other Pacific Islander individuals.

**Figure 2.**
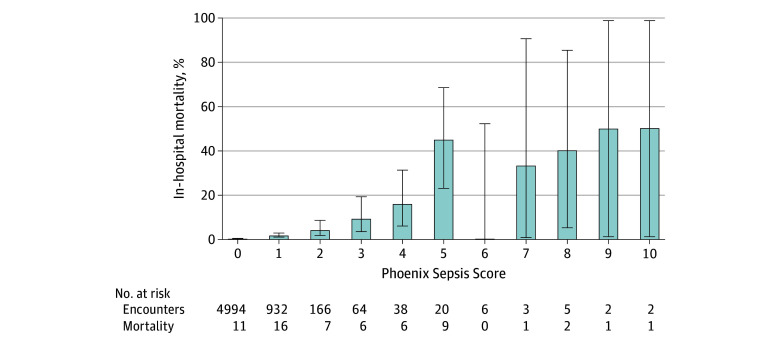
In-Hospital Mortality Associated With the Phoenix Sepsis Score in Patients With Suspected Community-Acquired Sepsis Bar indicates the median and whiskers the 95% CI.

Increasing Phoenix Sepsis Score was associated with a higher risk of death (Figure 2). In this cohort of patients with suspected community-acquired sepsis, 5910 of 6258 patients (94.4%) had a Phoenix Sepsis Score less than 2, in whom there were 27 of 60 (45.0%) in-hospital deaths.

Comparison of the SENTINEL and Phoenix patient cohorts are shown in Table 2. In-hospital mortality and death or ECLS within 72 hours in those meeting Phoenix criteria for sepsis were similar between the cohorts. The test characteristics between the cohorts (AUPRC, AUROC, sensitivity, and positive predictive value) were similar for predicting in-hospital mortality and death or ECLS within 72 hours. In this population, the worst Phoenix Sepsis Score calculated over the first 24 hours of hospitalization had an area under the precision recall curve of 0.17 (95% CI, 0.07-0.28) for predicting in-hospital mortality and 0.23 (95% CI, 0.11-0.36) for predicting death or ECLS within 72 hours. The Phoenix sepsis criteria had a sensitivity of 55.0% (95% CI, 41.6%-67.9%) and positive predictive value (PPV) of 10.8% (95% CI, 7.6%-14.9%) for in-hospital mortality and sensitivity of 77.8% (95% CI, 60.8%-89.9%) and PPV of 9.2% (95% CI, 6.2%-13.0%) for death or ECLS within 72 hours. Complete test characteristics of the Phoenix sepsis criteria for predicting in-hospital death and death or ECLS within 72 hours in the SENTINEL cohort are shown in eTable 5 in Supplement 1. The test characteristics of the original Phoenix cohort, restricted to data from high-income countries, are shown in eTable 6 in Supplement 1. Clinical vignettes of patients who did not meet the Phoenix sepsis criteria but died or required ICU admission for septic shock are described in the eAppendix in Supplement 1.

**Table 2.** Comparison of Phoenix Sepsis Score and Sepsis Criteria for Predicting In-Hospital Mortality and Death or ECLS Within 72 Hours Between SENTINEL and Original Phoenix Cohorts

Outcome	Test characteristic (95% CI)	
	SENTINEL	Phoenix validation
**Cohort characteristics**		
Met Phoenix Criteria for sepsis, No.	306	11792
In-hospital mortality, No. (%)	33 (10.8)	1167 (9.8)
Death or ECLS within 72 h, No. (%)	28 (9.2)	776 (6.6)
**Test characteristics for predicting in-hospital mortality**		
AUPRC	0.17 (0.07-0.28)	0.17 (0.04-0.26)
AUROC	0.75 (0.69-0.82)	0.75 (0.74-0.76)
Sensitivity, %	55.0 (41.6-67.9)	56.5 (54.3-58.7)
PPV, %	10.8 (7.6-14.9)	9.9 (9.4-10.5)
**Test characteristics for predicting death or ECLS within 72 h**		
AUPRC	0.23 (0.11-0.36)	0.24 (0.12-0.37)
AUROC	0.87 (0.80-0.94)	0.81 (0.80-0;82)
Sensitivity, %	77.8 (60.8-89.9)	68.1 (65.3-70.8)
PPV, %	9.2 (6.2-13.0)	6.6 (6.2-7.0)

Abbreviations: AUPRC, area under the precision recall curve; AUROC, area under the receiver operating characteristics curve; PPV, positive predictive value.

## Discussion

In this external validation study of the Phoenix Sepsis Score and sepsis criteria in a cohort of children with suspected community-acquired sepsis from Australia and New Zealand, the Phoenix Sepsis Score had similar performance to that in the original derivation and validation cohorts. Due to the infrequency of the primary and secondary outcomes, our study had wide confidence intervals for diagnostic accuracy parameters. The large sample size in the original Phoenix cohorts resulted in more precise estimates of test characteristics. In both cohorts, an increasing Phoenix Sepsis Score was associated with a higher risk of in-hospital mortality and a higher risk of death or ECLS within 72 hours. This confirms the criterion validity, reliability, and generalizability of the Phoenix Sepsis Score for predicting in-hospital mortality in a different patient population from the original derivation and validation cohort.

Overall, we found the Phoenix sepsis criteria had moderate performance for predicting in-hospital mortality, with a high proportion of missed deaths. The Phoenix sepsis criteria had better performance for predicting death or ECLS within 24 hours. We interpret this finding as an indicator that early death or ECLS was more likely to be attributable to sepsis.^13^ The limited ability of the Phoenix sepsis criteria to predict in-hospital mortality may have resulted from clinical deterioration more than 24 hours from hospitalization (see clinical vignette 1 in the eAppendix in Supplement 1); death not attributable to sepsis (see clinical vignette 2 in the eAppendix in Supplement 1); or death due to progression of underlying disease rather than death due to sepsis (see clinical vignette 3 in the eAppendix in Supplement 1); and lastly, any effort to predict in-hospital mortality is confounded by clinical efforts to prevent death through the use of antimicrobials and organ support therapies.

Our cohort of children had fewer missing data than in the original Phoenix cohort, including most physiological variables and serum lactate. Coagulation variables, such as international normalized ratio and D-dimer, were missing in a high proportion of patients in both cohorts. Missing data were assumed to be normal and did not contribute to the overall Phoenix Sepsis Score. This assumption may be incorrect, as evidenced by the higher proportion of missing data in the cohort from low- and middle-resource settings in the original Phoenix cohort despite having higher in-hospital mortality.^5^

The process followed by the sepsis definition task force in developing the Phoenix Sepsis Score and sepsis criteria was comprehensive and exhaustive; using a global survey of clinicians,^14^ a systematic review and meta-analysis,^15^ and a large international derivation and validation study.^5^ The definition was designed to reliably identify children with sepsis for the purposes of clinical care, benchmarking, quality improvement, epidemiology, and research.^16^ The task force did not intend the sepsis definition to be used for sepsis screening or early identification of children with suspected sepsis. The Phoenix criteria are therefore not intended to help determine when and in whom to initiate treatment for sepsis. Our data and that from the original Phoenix validation cohort support the use of the Phoenix criteria for benchmarking clinical care between populations, as a standard that can be used to report sepsis epidemiology, and as a method for comparing similar populations over time for quality improvement purposes. Our data and that from the original Phoenix validation highlight some limitations of the Phoenix sepsis criteria: (1) most children (>95%) did not meet the Phoenix sepsis criteria, indicating a lack of discriminative ability for a large proportion of children hospitalized with suspected sepsis and an underestimate of the burden of suspected sepsis on the health care system; (2) the Phoenix sepsis criteria are determined as the worst score after the first 24 hours of hospitalization, limiting their utility for clinical care or research within the first 24 hours of hospitalization; and (3) the criteria did not identify nearly half of the children who died in hospital, indicating a lack of sensitivity and predictive ability for overall mortality. These limitations should be taken into consideration when applying the Phoenix Sepsis Score and sepsis criteria in clinical practice.

### Limitations

This study has limitations. Our data are limited to 2 high-income countries, and our inclusion criteria were somewhat different from the original Phoenix cohort. Our study took place over the COVID-19 pandemic, when most hospitalized children had COVID-19 screening even when asymptomatic, confounding the use of microbiological testing as an indicator of suspected infection. The original Phoenix study used data collected before the COVID-19 pandemic, making microbiological testing a more valid indicator of suspected infection. As a result, baseline characteristics in the SENTINEL cohort differed in some respects from the original Phoenix derivation and validation cohorts. Despite these differences in inclusion criteria and some baseline characteristics, our populations were remarkably similar in terms of ICU admission rates, baseline mortality, and death or ECLS within 72 hours. We did not find a difference in patient characteristics when either of our inclusion criteria were applied in isolation.

## Conclusions

In conclusion, the Phoenix Sepsis Score and sepsis criteria in this cohort of children from Australia and New Zealand performed similarly to the original retrospective derivation and validation cohorts. Both cohorts had a high proportion of missing data. Higher Phoenix Sepsis Scores were associated with a higher risk of in-hospital mortality, although the Phoenix sepsis criteria did not identify approximately half of the children who died in hospital.

## Notes

Supplement 1. **eTable 1. **Enrolling sites with duration and numbers enrolled **eTable 2. **Inclusion and exclusion criteria of the SENTINEL cohort compared with the original Phoenix cohort **eTable 3. **Patient characteristics comparing the two SENTINEL inclusion criteria **eTable 4. **Data missingness in the SENTINEL and original Phoenix cohorts **eTable 5. **Phoenix sepsis criteria characteristics (SENTINEL data) **eTable 6. **Phoenix sepsis criteria characteristics (original Phoenix data restricted to high-resource settings) **eAppendix. **Clinical vignettes

Supplement 2.
Nonauthor Collaborators

Supplement 3.
Data Sharing Statement
