# Supplementary material for: External Validation of the Phoenix Sepsis Score in Children With Suspected Community-Acquired Sepsis
Source: JAMA Netw Open. 2025 Mar 21;8(3):e251412. doi: 10.1001/jamanetworkopen.2025.1412 (PMC11929021; doi:10.1001/jamanetworkopen.2025.1412)
Supplement: Supplement 1. — eTable 1. Enrolling sites with duration and numbers enrolled eTable 2. Inclusion and exclusion criteria of the SENTINEL cohort compared with the original Phoenix cohort eTable 3. Patient characteristics comparing the two SENTINEL inclusion criteria eTable 4. Data missingness in the SENTINEL and original Phoenix cohorts eTable 5. Phoenix sepsis criteria characteristics (SENTINEL data) eTable 6. Phoenix sepsis criteria characteristics (original Phoenix data restricted to high-resource settings) eAppendix. Clinical vignettes [file jamanetwopen-e251412-s001.pdf]

Supplemental Online Content

Long E, Borland ML, George S, et al; Paediatric Research in Emergency Departments International Collaborative (PREDICT) Network. External validation of the Phoenix Sepsis Score in children with suspected community-acquired sepsis. *JAMA Netw Open.* 2025;8(3):e251412. doi:10.1001/jamanetworkopen.2025.1412

eTable 1. Enrolling sites with duration and numbers enrolled

eTable 2. Inclusion and exclusion criteria of the SENTINEL cohort compared with the original Phoenix cohort

eTable 3. Patient characteristics comparing the two SENTINEL inclusion criteria

eTable 4. Data missingness in the SENTINEL and original Phoenix cohorts

eTable 5. Phoenix sepsis criteria characteristics (SENTINEL data)

eTable 6. Phoenix sepsis criteria characteristics (original Phoenix data restricted to high-resource settings)

eAppendix. Clinical vignettes

This supplemental material has been provided by the authors to give readers additional information about their work.

© 2025 Long E et al. *JAMA Network Open*.

eTable 1. Enrolment dates and numbers by study site.

**Study site**	**State / territory**	**Annual Emergency Department census**	**Enrolment dates**	**Total number of enrolments**
Australian sites				
Monash Medical Centre	Victoria	42,000	Sept 2021– July 2023	782
Children’s Hospital at Westmead	New South Wales	64,000	May 2022– July 2023	894
The Royal Children’s Hospital	Victoria	89,000	April 2021 – December 2023	1471
Queensland Children’s Hospital	Queensland	76,000	May 22– July 2023	622
Perth Children’s Hospital	Western Australia	71,000	Sept 2021– July 2023	644
Women’s and Children’s Hospital	South Australia	54,000	Oct 2021– July 2023	693
The Royal Darwin Hospital	The Northern Territory	20,000	Feb 2022– July 2023	111
Gold Coast University Hospital	Queensland	32,000	Nov 2021– July 2023	476
Townsville Hospital	Queensland	20,000	July 2023 – December 2023	53
New Zealand sites				
Kidz First Hospital	Middlemore	32,000	Oct 2022 – December 2023	230
Starship Hospital	Auckland	39,000	Nov 2022– December 2023	282

© 2025 Long E et al. *JAMA Network Open*.

eTable 2. Inclusion and Exclusion Criteria for SENTINEL and Phoenix cohorts

SENTINEL cohort inclusion criteria	Original Phoenix cohort inclusion criteria
• Emergency department presentation<br>• Up to 18 years of age<br>• Admission to the hospital<br>• Treatment with parenteral antibiotics<br>• Provisional diagnosis of sepsis OR treatment for sepsis*	• Emergency department, inpatient unit, or intensive care unit presentation<br>• Younger than 18 years of age<br>• Suspected infection (receipt of systemic antimicrobials and microbiological testing within 24 hours of encounter)
**SENTINEL cohort exclusion criteria**	**Original Phoenix cohort exclusion criteria**
• Patients not admitted through the emergency department<br>• Interhospital transfers from a hospital ward to the emergency department<br>• Patients with trauma	• Newborns<br>• Post conceptual age <37 weeks

*treatment for sepsis: administration of one or more fluid bolus (fixed volume of fluid administered over <30 minutes to treat impaired perfusion, not dehydration)

© 2025 Long E et al. *JAMA Network Open*.

eTable 3. Comparison of patient characteristics by inclusion criteria.

	SENTINEL cohort	Inclusion Criteria	
		Provisional diagnosis of sepsis	Treatment for sepsis
	(N=6,232)	(n=3,772)	(n=4,629)
**Age at presentation (years)**, median (IQR)	2.1 (0.3-7.1)	1.3 (0.1-5.5)	2.4 (0.4-7.7)
**Sex**, n(%)			
Female	2,830 (45.5)	1,688 (44.7)	2,145 (46.3)
Male	3,386 (54.5)	2,084 (55.2)	2,484 (53.6)
Other	2 (<0.1)	1 (<0.1)	2 (<0.1)
**Ethnicity**, n(%)			
Indigenous (Aboriginal and Torres Strait Islander, Māori, American Indian, Alaskan Native, Native Hawaiian, other Pacific Islander)	599 (9.6)	385 (10.2)	498 (10.8)
**Comorbidities per PCCC**, n(%)			
1 PCCC	1,168 (18.7)	664 (17.6)	880 (19.0)
≥ 2 PCCC	1,345 (21.6)	770 (20.4)	941 (20.3)
**ICU admission**, n(%)	1,080 (17.4)	609 (16.2)	798 (17.3)
**Outcomes**, n(%)			
In-hospital mortality	60 (1.0)	36 (1.0)	54 (1.2)
Death or extracorporeal life support within 72 hours	36 (0.6)	22 (0.6)	34 (0.7)

NB- 2161 patients met inclusion criteria for both provisional diagnosis of sepsis and treatment for sepsis

IQR, inter-quartile range. PCCC, pediatric complex chronic condition. ICU, intensive care unit.

© 2025 Long E et al. *JAMA Network Open*.

eTable 4. Organ dysfunction subscore data missingness over the first 24-hours of hospitalisation.

Organ dysfunction subscore	% missing data over time					
	Within 6 hours		Within 12 hours		Within 24 hours	
	SENTINEL Cohort	Phoenix Cohort	SENTINEL Cohort	Phoenix Cohort	SENTINEL Cohort	Phoenix Cohort
**Respiratory**						
SpO2	3.1	48	3.1	41	3.1	37
PaO2	-	97	-	95	-	92
FiO2	95.6	80	95.1	77	94.8	75
Any respiratory support	3.5	N/R	3.9	N/R	4.3	N/R
Mechanical ventilation	0.8	0	0.8	0	0.8	0
**Cardiovascular**						
MAP	23.7	13	22.8	12	22.3	12
Lactate	23.1	90	23.1	88	23.1	84
Any vasoactive	0.8	0	0.8	0	0.8	0
**Coagulation**						
Platelet count	9.5	40	9.5	39	9.5	37
INR	89.2	90	89.2	88	89.2	84
D-dimer	-	98	-	97	-	96
Fibrinogen	89.1	96	89.1	95	89.1	93
**Neurological**						
Glasgow Coma Scale score	15.8	55	15.4	52	15.2	48
Pupils	1.2	80	1.2	78	1.2	76

N/R, not reported; -, data not recorded; SpO2, pulse oximeter oxygen saturation; PaO2, arterial oxygen partial pressure; FiO2, fraction of inspired oxygen; MAP, mean arterial pressure; INR, international normalized ratio. NB: missingness for the Phoenix cohort is reported for higher resource settings.

© 2025 Long E et al. *JAMA Network Open*.

eTable 5. Test characteristics of the Phoenix criteria for sepsis diagnosis for predicting death or death / requirement for extracorporeal life support within 72 hours when applied to the SENTINEL cohort.

	AUPRC	AUROC	Sens (%)	Spec (%)	PPV (%)	NPV (%)
In-hospital mortality; n=60	0.17 (0.07-0.28)	0.75 (0.69-0.82)	55.0 (41.6-67.9)	95.6 (95.0-96.1)	10.8 (7.6-14.9)	99.5 (99.3-99.7)
Death or extracorporeal life support within 72 hours; n=36	0.23 (0.11-0.36)	0.87 (0.80-0.94)	77.8 (60.8-89.9)	95.5 (95.0-96.0)	9.2 (6.2-13.0)	99.9 (99.7-99.9)

Abbreviations: AUPRC, area under the precision recall curve; AUROC, area under the receiver operating characteristics curve; Sens, sensitivity; Spec, specificity; PPV, positive predictive value; NPV, negative predictive value.

© 2025 Long E et al. *JAMA Network Open*.

eTable 6. Test characteristics of the Phoenix criteria from the original derivation and internal validation cohorts, restricted to high-resource settings.

	AUROC	Sens (%)	Spec (%)	PPV (%)	NPV (%)
In-hospital mortality; n=60	0.81 (0.80-0.83)	69.2 (66.3-72.0)	93.4 (93.2-93.5)	7.1 (6.6-7.6)	99.8 (99.7-99.8)
Death or extracorporeal life support within 72 hours; n=36	0.92 (0.90-0.93)	90.0 (87.4-92.3)	93.3 (93.1-93.4)	5.5 (5.0-5.9)	99.9 (99.9-100.0)

AUROC, area under the receiver operating characteristics curve; Sens, sensitivity; Spec, specificity; PPV, positive predictive value; NPV, negative predictive value. NB: area under the precision recall curve (AUPRC) could not be calculated for aggregate data.

© 2025 Long E et al. *JAMA Network Open*.

eAppendix. Clinical vignettes for patients who did not fulfill Phoenix sepsis criteria.*

Case #1, intensive care unit admission for noradrenaline (norepinephrine) infusion:
A previously healthy 9-year-old girl presents to a study site emergency department with abdominal pain. She is noted to have a temperature of 38.2ºC, tachycardia, an oxygen saturation of 93% in room air, grunting respirations, and a peritonitic abdomen. Computed tomography scan of her abdomen reveals perforated appendicitis. She has intravenous access obtained, is started on broad spectrum antibiotics, receives fluid resuscitation as per local guidelines, and admitted to a surgical ward.

Phoenix sepsis score: Within the first 24-hours, 0 respiratory points (no hypoxemia or respiratory support), 0 cardiovascular points (no hypotension, lactate <5mmol/L), 0 coagulation points (normal platelet count, INR, and fibrinogen), 0 neurological points (Glasgow Coma Scale score 15, pupils reactive). On the 8-item Phoenix sepsis score, she would have scored +1 for elevated creatinine (108umol/L).

Phoenix sepsis criteria: The patient has suspected infection and 0 points on the Phoenix sepsis score, so does not meet the criteria for sepsis or septic shock.

The following day, she is found intra-operatively to have perforated appendicitis sealed by omentum with no peritoneal soiling. Post operatively she develops hypotension refractory to fluid resuscitation and is commenced on a noradrenaline (norepinephrine) infusion and transferred to the Intensive Care Unit. Her blood culture is positive for *Escherichia coli*. Her noradrenaline was weaned over 48 hours and she remains hospitalised for 9 days total for treatment with intravenous antibiotics.

Case #2, death in hospital not likely attributable to sepsis:
A 3-month old boy presents to a study site emergency department with poor feeding and vomiting after having recent immunisations. He was born at 39 weeks by emergency caesarean section for breech presentation following an uncomplicated pregnancy. He had 10-day newborn unit admission for poor weight gain. During his initial assessment he had normal vital signs but continued vomiting. He was admitted to the hospital with a diagnosis of urosepsis and treated with intravenous antibiotics and intravenous fluids.

Phoenix sepsis score: Within the first 24 hours, 0 respiratory points (no hypoxemia or respiratory support), 0 cardiovascular points (no hypotension, lactate <5mmol/L), 0 coagulation points ((normal platelet count, INR, and fibrinogen), 0 neurological points (Glasgow Coma Scale score 15, pupils reactive).

Phoenix sepsis criteria: The patient has suspected infection and 0 points on the Phoenix sepsis score, so does not meet the criteria for sepsis or septic shock.

On day 2 of his admission he deteriorates with seizure activity and apnea and is transferred to the Intensive Care Unit for respiratory support (via high-flow nasal cannula). His antimicrobial cover is broadened to include cover for central nervous system infection (bacterial and viral). He develops nephrotic syndrome and refractory seizures, and is diagnosed with a severe phenotype of a rare progressive neurodegenerative disorder. He is palliated and dies 17 days following admission.

Case #3, death in hospital likely attributable to sepsis:

© 2025 Long E et al. *JAMA Network Open*.

A 12-year-old boy with relapsed T-cell acute lymphoblastic leukemia following failed bone marrow transplant presents to a study site emergency department with a temperature of 38°, tachycardia, and mucositis. Blood pressure with an oscillometric device is 111/65mmHg (mean blood pressure of 80mmHg). His indwelling central venous access device is accessed, he is started on broad spectrum antibiotics and antifungals, administered fluid resuscitation as per local guidelines, and cultures of blood and urine are sent. He is commenced on a hydromorphone infusion for oral pain. A complete blood count reveals mild anaemia, platelets of 75K/uL, and a normal white cell count.

Phoenix sepsis score: Within the first 24 hours, 0 respiratory points (no hypoxemia or respiratory support), 0 cardiovascular points (no hypotension, lactate<5mmol/L), +1 coagulation point (for low platelet count), 0 neurological points (Glasgow Coma Scale score of 15 and reactive pupils).

Phoenix sepsis criteria: The patient has suspected infection and 1 point on the Phoenix sepsis score, with 0 cardiovascular points, so does not meet criteria for sepsis or septic shock.

He is admitted to the oncology ward for ongoing care. Despite maximal treatment for underlying infection, he has a respiratory deterioration on day 4 of admission. He is admitted to the Paediatric Intensive Care Unit, and in consultation with his family, the decision for palliation is made. He dies 5 days following admission.

*Cases were anonymized to maintain patient confidentiality.

© 2025 Long E et al. *JAMA Network Open*.
