# Supplementary material for: External Validation of the Phoenix Sepsis Score in Children With Suspected Community-Acquired Sepsis
Source: JAMA Netw Open. 2025 Mar 21;8(3):e251412. doi: 10.1001/jamanetworkopen.2025.1412 (PMC11929021; doi:10.1001/jamanetworkopen.2025.1412)
Supplement: Supplement 2. — Nonauthor Collaborators [file jamanetwopen-e251412-s002.pdf]

Supplemental Online Content: Nonauthor Collaborators
*First name, last name, and suffix (if applicable) are required and will appear in PubMed.

*Group Name(s): Paediatric Research in Emergency Departments International Collaborative (PREDICT) Network								
***First Name and Middle Initial(s)**	***Last Name**	***Suffix (eg, Jr, III)**	Academic Degrees	Institution	Location (city, state/province, country)	Role or Contribution, eg, chair, principal investigator	Group (if more than 1 Group listed in the byline) and/or Subgroup (eg, Steering Committee)	ORCID
Catherine L	Wilson	Ms	MPH; Ms Eval. Assess.	Murdoch Children's Research Institute	Parkville, Victoria, Australia	Member of Steering Group	PREDICT	(https://orcid.org/0000-0001-5893-4226)
