# Supplementary material for: External Validation of the Phoenix Sepsis Score in Children With Suspected Community-Acquired Sepsis
Source: JAMA Netw Open. 2025 Mar 21;8(3):e251412. doi: 10.1001/jamanetworkopen.2025.1412 (PMC11929021; doi:10.1001/jamanetworkopen.2025.1412)
Supplement: Supplement 3. — Data Sharing Statement [file jamanetwopen-e251412-s003.pdf]

Data Sharing Statement

Long. External Validation of the Phoenix Sepsis Score in Children With Suspected Community-Acquired Sepsis. *JAMA Netw Open*. Published March 21, 2025.
doi:10.1001/jamanetworkopen.2025.1412

Data
Data available: Yes
Data types: Deidentified participant data
How to access data: Data will be provided upon written request to the corresponding author (elliot.long@rch.org.au) and provided within the constraints of ethics and regulatory requirements.
When available: beginning date: 11-29-2025

Supporting Documents
Document types: None

Additional Information
Who can access the data: researchers whose proposed use of the data has been approved
Types of analyses: for any purpose
Mechanisms of data availability: With approval of a proposal
Any additional restrictions: ethical and regulatory constraints where applicable
